# Green Extraction of Phenolic Compounds From *Pistacia lentiscus* Leaves Coupled With LC‐MS Characterization and Structure‐Based Enzyme Inhibition Analysis

**DOI:** 10.1002/cbdv.71831

**Published:** 2026-10-12

**Authors:** Sidali Zaidi, Naima Saidene, Radia Kadi, Nadjet Benaida‐Debbache

**Affiliations:** ^1^ Centre de Recherche en Technologies Agro‐Alimentaires Campus Universitaire Bejaia Algeria; ^2^ Faculté Des Sciences de La Nature Et de La Vie, Laboratoire de Biochimie Appliquée Université de Bejaia Bejaia Algeria; ^3^ Faculté Des Sciences de La Nature Et de La Vie Laboratoire de Biomathématique, Biophysique, Biochimie Et Scientométrie (3BS) Université de Bejaia Bejaia Algeria

**Keywords:** enzymatic activities, lactic acid, molecular docking, *P. lentiscus*, ultrasound‐assisted extraction

## Abstract

Acetylcholinesterase (AChE) and urease are critical enzymes involved in various pathological conditions associated with cellular and tissue damage. Natural products are increasingly explored as safer alternatives to synthetic inhibitors. This study evaluates the phytochemical composition and enzyme inhibitory potential of *Pistacia lentiscus* leaves using a green extraction approach. Bioactive compounds were extracted via lactic acid‐assisted ultrasound, and the crude extract was characterized for total phenolics, flavonoids, LC‐MS profile, and in vitro inhibition of acetylcholinesterase and urease. Molecular docking was performed to investigate ligand–enzyme interactions. The extract contained 142.13 ± 0.06 mg GAE/g DW of polyphenols and 16.17 ± 0.02 mg QE/g DW of flavonoids. LC‐MS analysis identified phenolic acids, phenolic glycosides, coumarins, and multiple flavonoid subclasses, including flavonols, flavanols, flavanones, flavones, and flavonolignans. The extract exhibited significant inhibition of both enzymes. Docking studies revealed key interactions, such as hydrogen bonds, π‐alkyl, and π‐sigma, within the active sites, with hesperidin and verbascoside showing the strongest binding affinities (acetylcholinesterase: –10.1 and –10.2 kcal/mol; urease: –9.6 and –9.2 kcal/mol, respectively). These results demonstrate that ultrasound‐assisted lactic acid extraction efficiently isolates phenolic compounds from *P. lentiscus*, and the identified metabolites may serve as promising templates for the development of potent enzyme inhibitors.

## Graphical abstract

Ultrasound‐assisted lactic acid extraction of *Pistacia lentiscus* leaves yielded phenolic‐rich extracts characterized by LC‐MS. The extract showed strong in vitro inhibition of acetylcholinesterase and urease. Docking highlighted hesperidin and verbascoside as key ligands, supporting this green extraction approach as a source of potent natural enzyme inhibitors.

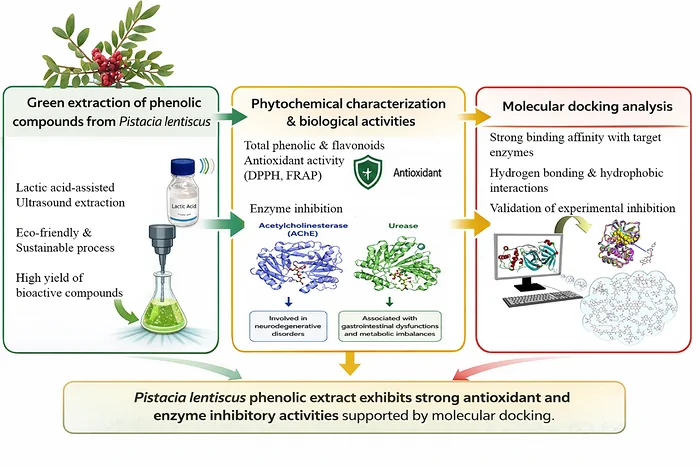

## 1 Introduction

Acetylcholinesterase (AChE) and urease are key enzymes implicated in diverse pathological processes, including neurodegenerative disorders, gastrointestinal dysfunctions, and metabolic imbalances, where their dysregulation can lead to cellular and tissue damage [1, 2]. The inhibition of these enzymes is therefore a therapeutic target of considerable interest. While several synthetic inhibitors are available, concerns regarding their toxicity, side effects, and environmental impact have prompted the search for safer, bio‐based alternatives.

Natural products, particularly plant‐derived phenolics and flavonoids, have emerged as promising enzyme modulators due to their structural diversity, biocompatibility, and multifunctional bioactivities. The Mediterranean shrub *Pistacia lentiscus* L. (Anacardiaceae) is a well‐recognized source of antioxidant and bioactive secondary metabolites, including phenolic acids, flavonoids, coumarins, and glycosides, which are associated with antioxidant, anti‐inflammatory, antimicrobial, and neuroprotective properties [3, 4, 5]. Despite this potential, systematic studies combining sustainable extraction methods, chemical profiling, and evaluation of enzyme inhibitory activity remain limited.

Green extraction technologies, such as ultrasound‐assisted extraction (UAE) using bio‐based solvents, offer environmentally friendly alternatives to conventional methods, providing efficient recovery of polar phytochemicals while minimizing solvent usage and thermal degradation. Lactic acid, a biodegradable and biocompatible organic acid, has recently been shown to enhance the solubilization of phenolics and stabilize labile compounds during extraction [5, 6].

In this context, the present study investigates the use of lactic acid‐assisted ultrasound extraction to recover bioactive compounds from *P. lentiscus* leaves. Extracts were quantitatively analyzed for total phenolics and flavonoids, chemically characterized using liquid chromatography‐mass spectrometry (LC‐MS), and evaluated for in vitro acetylcholinesterase and urease inhibitory activities. Molecular docking studies were further conducted to elucidate the interactions between major phenolic constituents and target enzymes, providing mechanistic insight into their inhibitory potential. This integrated approach aims to highlight both the phytochemical richness of *P. lentiscus* and its potential as a source of natural enzyme inhibitors for biomedical application.

## 2 Material and Method

### 2.1 Instrumentation

Electric grinder IKA M20 WERKE (United States), analytical balance RADWAG AS 220.R2 (Poland), magnetic stirrer Velp Scientifica AM4 (Italy), water bath Memmert WPE (Germany), ultrasonic bath TIERRATECH LT‐100 PRO 100 W/40 kHz (Mexico), refrigerated centrifuge Sigma 2–16 PK (Germany), Vortex V‐1 Plus BOECO (Germany), Orion 420A pH mètre—Gemini BV (Germany), microplate reader BioTek Synergy HTX Agilent BioTek S1LFA‐SN (Santa Clara, United States), microplate incubator BOECO PST‐60 HL (Germany), and LC‐MS Shimadzu LCMS 8040 (Japanese).

### 2.2 Chemical Product

All reagents and chemicals used were of analytical grade: ethanol, l‐lactic acid, formic acid, acetonitrile, hypochlorite, 2,2‐diphenyl‐1‐picrylhydrazyl (DPPH), acetylcholine esterase, acetylcholine iodide, urease, urea, Na_2_CO_3_, hydroxide sodium, Coomassie brilliant blue G‐250, phosphoric acid, trichloro‐acetic acid (TCA), Na_2_HPO_4_, NaH_2_PO_4_ from Sigma Aldrich (Germany). AlCl_3_, bovine serum albumin (BSA), sodium acetate, from Alpha Chemica (India). Gallic acid, quercetin, ascorbic acid, citric acid, DTNB, Folin–Ciocalteau reagent was obtained from Biochem Chemopharma (USA). Potassium ferrocyanide, ferrous chloride (FeCl_2_), ferric chloride (FeCl_3_), phenol, nitruprusside from Panreac (Spain).

### 2.3 Plant Material

*Pistacia lentiscus* is a well‐known medicinal plant in the Algerian pharmacopoeia. The leaves were collected in early September 2025 in a rural area of Marj Waman, specifically in the Tizi Neftah forest (GPS: 36.644°N, 4.921°E Bejaïa province, Algeria) located in the Bejaia province of Algeria, benefiting from a privileged location along the Mediterranean coast. The geographical coordinates of this city are approximately 36.75° north latitude and 5.07° east longitude.

The collected sample is carefully washed with running water and then dried in the oven at 40°C for 2 days. Subsequently, the dried sample is ground into a powder using an electric grinder and then sieved to achieve a homogeneous powder with a diameter of less than 63 µm. Finally, it is stored in the dark at −20°C until use.

The identification of the plant material was performed by Professor Sahnoune at the Botany Laboratory, University of Béjaïa, Algeria. A voucher herbarium specimen (No. 970704) has been deposited at the National Institute of Agronomy, Algiers, Algeria, and is available for future reference and verification.

### 2.4 Extraction Procedure

The extraction of phytoconstituents from *P. lentiscus* leaves was carried out using a sustainable lactic acid‐mediated process. In this study, powdered *P. lentiscus* leaves were added to a 63.5% lactic acid solution at a ratio of 1:10 (w/w). The mixture was then subjected to ultrasound‐assisted extraction in a water bath for 30 min at 45°C to enhance the release of bioactive compounds. These optimized conditions have been previously validated to maximize the recovery of phenolic compounds, particularly from *P. lentiscus* matrices. Subsequently, the lactic acid mixture was centrifuged to remove any residual powder. The clarified extract was collected and stored at –4°C prior to subsequent analyses, including quantitative determination of bioactive compounds, LC‐MS profiling, and enzymatic activity assays, whereas the extraction solvent (lactic acid) served as the negative control [5].

### 2.5 Spectrophotometric Analysis

The crude extract of P. lentiscus was characterized using a UV–visible spectrophotometer connected to a microcomputer equipped with UV‐Probe software, which enabled the recording and visualization of the spectra. The extract was scanned over a wavelength range of 190–1000 nm using a 1 cm path‐length quartz cuvette.

### 2.6 Characterizations LC‐MS

The analysis of the lactic acid‐enriched sample was performed using LC‐MS (Shimadzu LCMS 8040) coupled with Q‐3 mass spectrometry in both negative and positive electrospray ionization modes. Chromatographic separation was carried out using a C18 XSelect column (150 mm × 2.1 mm, 3.5 µm) in reverse phase. The mobile phase consisted of a mixture of acidified water (0.1% formic acid) (eluent A) and acetonitrile (eluent B) with the following gradient profile: 0–3 min, 10% B; 3–6 min, 30% B; 6–10 min, 50% B; 10–15 min, 75% B; 15–20 min, 100% B; 20–24 min, 50% B; 24–28 min, 30% B; 28–40 min, 10% B. The HPLC technique was used at a column oven temperature of 40°C, a flow rate of 0.25 mL/min, and a sample volume of 10 µL. Ions were identified by their exact mass (*m*/*z*), isotopic ratio, and fragmentation pattern [5].

### 2.7 Measurement of Bioactive Compounds

The total polyphenol content (TPC) of the lactic acid extract was determined using the Folin–Ciocalteu colorimetric method, as described by Moldovan et al. [7]. Briefly, 20 µL of the lactic acid‐enriched extract was mixed with 100 µL of diluted Folin–Ciocalteu reagent (1:10, v/v) and incubated at room temperature for 2–3 min. Subsequently, 80 µL of a 7.5% (w/v) sodium carbonate (Na_2_CO_3_) solution was added, and the reaction mixture was further incubated for 30 min. The absorbance was then measured at 760 nm using a UV–visible spectrophotometer. The TPC was calculated from a gallic acid calibration curve prepared under identical conditions and expressed as milligrams of gallic acid equivalents per gram of dry weight (mg GAE/g DW).

The total flavonoid content (TFC) was quantified according to the aluminum trichloride (AlCl_3_) colorimetric method described by Djeridane et al. [8]. In this assay, 100 µL of the lactic acid extract was mixed with 100 µL of a 2% (w/v) AlCl_3_ methanolic solution. After incubation for 15 min at room temperature, the absorbance was recorded at 430 nm. The flavonoid content was determined using a quercetin calibration curve established under the same experimental conditions and expressed as milligrams of quercetin equivalents per gram of dry weight (mg QE/g DW).

### 2.8 Evaluation of Antioxidant Potential

The antioxidant activity of the extracts was evaluated using both DPPH• radical scavenging and reducing power assays. The DPPH• scavenging capacity was assessed according to the method described by Ambarwati et al. [9], in which 180 µL of an ethanolic DPPH• solution was mixed with 20 µL of the extract or standard and incubated at 37°C for 30 min. The decrease in absorbance was measured at 515 nm, and the antioxidant activity was expressed as mmol Trolox equivalents per 100 g of dry weight (mmol TE/100 g DW).

In parallel, the reducing power of the extracts was determined following the procedure reported by Menakh et al. [10]. Briefly, 10 µL of extract or standard was mixed with 40 µL of phosphate buffer and 50 µL of 1% K_3_Fe(CN)_6_, then incubated at 50°C for 20 min. After incubation, 50 µL of 10% trichloroacetic acid were added, followed by 40 µL of distilled water and 10 µL of 0.1% FeCl_3_. The absorbance was recorded at 700 nm, and the reducing power was expressed as mg ascorbic acid equivalents per gram of dry weight (mg AAE/g DW).

### 2.9 Anticholinesterase Assay

The evaluation of the anticholinesterase activity was performed according to the protocol of Ellman et al. [11]. In a microplate, 20 µL of the lactic acid extract or standard (gallic acid) at different dilutions were added to 130 µL of enzyme (3 U in the buffer). After 5 min of incubation, 30 µL of DTNB (10 mM, with and 50 mM sodium acetate in phosphate buffer 0.2 M, pH 7.4) and 20 µL of acetylcholine iodide (15 mM) were added, and the mixture was incubated for an additional 5 min. Finally, the absorbance of the reaction mixture was recorded at 405 nm. The activity of the extract is expressed as a percentage of inhibition determined according to the following equation (Equation 1):

(1) $$\%\;=\frac{\mathrm{Ab}s_{\left(\mathrm{Ctrol}\right)}-\mathrm{Ab}s_{\left(\mathrm{smpl}\right)}}{\mathrm{Ab}s_{\left(\mathrm{ctrol}\right)}}\;\times 100$$

where, Abs_(ctrol)_: control absorbance; Abs_(smpl)_: sample absorbance.

### 2.10 Antiurease Assay

The estimation of the antiurease activity was carried out according to the following protocol of Weatherburn [12]. A volume of 25 µL of urease (4 U in a 200 mM phosphate buffer, pH 7.4) was incubated with 15 µL of the lactic acid extract or standard (boric acid) at different dilutions for 15 min at 30°C. Then, 40 µL of urea at 100 mM was added to the reaction mixture, followed by incubation for 30 min at 30°C. After incubation, 50 µL of the phenol reagent (1% phenol + 0.005% nitroprusside) and 70 µL of the alkaline reagent (0.7% hypochlorite + 0.5% NaOH) were added. Finally, the absorbance was measured after 60 min at 630 nm and the results were expressed as percent inhibition (Equation (1)).

### 2.11 In Silico Study

An in silico study was carried out to elucidate the interaction patterns of bioactive compounds from the crude lactic acid extract within the catalytic cavities of acetylcholinesterase and urease. The phenolic compounds previously identified by LC‐MS analysis were selected as ligands for molecular docking simulations. Three‐dimensional structural data of the ligands were retrieved from the PubChem database in SDF format and subsequently converted to PDB format using PyMOL software to ensure compatibility with AutoDock Vina. The three‐dimensional crystal structures of acetylcholinesterase (PDB ID: 4PQE) and urease (PDB ID: 3LA4), with resolutions of 2.90 and 2.05 Å, respectively, were retrieved from the RCSB Protein Data Bank. Prior to molecular docking, protein structures were prepared using AutoDock Tools by removing crystallographic water molecules, adding polar hydrogen atoms, and assigning Kollman charges. These preprocessing steps ensured proper optimization of the macromolecular targets for subsequent virtual screening analyses. The binding affinity energy (Δ*G*, kcal/mol) of each compound, obtained from AutoDock Vina simulations, was further used to estimate the predicted inhibition constant (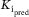), which was subsequently employed to calculate the corresponding p*K*_i_ value, defined as the negative base‐10 logarithm of the inhibition constant, according to the following equations (Equations (2 and 3)):

(2) $$K_{i_{\mathrm{pred}}}=e^{\Delta G/RT}$$

(3)

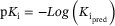

where ∆*G* is the binding affinity (kcal mol^−1^), *R* (gas constant) is 1.98 cal∗mol∗K^−1^, *T* (room temperature) is 298.15 Kelvin, and 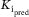 is the predicted inhibitory constant.

### 2.12 Statistical Data Analysis

All measurements were performed in triplicate, and the results are expressed as mean ± standard deviation. The data were subjected to analysis of variance (ANOVA) using the *F*‐test in R software, with the least significant difference (LSD) test, at a significance level of *p <* 0.05.

## 3 Results and Discussion

### 3.1 Spectral Analysis UV–Visible

The UV–visible spectral analysis of the crude extracts of *P. lentiscus* revealed the presence of intense absorption bands mainly distributed between 200 nm and 320 nm (Figure 1). This spectral region is characteristic of organic compounds containing aromatic rings and conjugated systems, where UV absorption arises predominantly from electronic transitions involving π–π* orbitals, as well as transitions associated with non‐bonding electron pairs of heteroatoms (*n*–σ* and *n*–π*). Given the complex chemical nature of crude plant extracts, the observed absorption profile reflects the cumulative contribution of multiple chromophoric systems.

**FIGURE 1:**
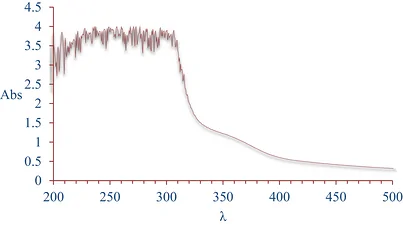
UV–Visible spectrum of the lactic acid extract of *Pistacia lentiscus*.

The recorded spectra can be primarily attributed to phenolic acids and flavonoid compounds, which are well known to dominate the secondary metabolite profile of *P. lentiscus*. Phenolic acids with a benzoic acid backbone typically exhibit absorption maxima in the 200–290 nm range, while flavonoids—particularly flavones and flavonols—display two characteristic absorption bands. Band II, generally observed between 250 nm and 285 nm, is associated with the benzoyl system (ring A–C conjugation), whereas Band I, appearing in the 320–385 nm region, corresponds to the cinnamoyl system involving extended conjugation between rings B and C. The predominance of absorption signals in the lower UV region suggests a higher relative abundance of phenolic acids and moderately conjugated flavonoids in the crude extract.

The optical density measurements enabled the determination of several absorption maxima (*λ*_max), with high absorbance values recorded at 236.5, 237.5, 256, 260, 268, 271, 273, and 276.5 nm. These *λ*_max values are consistent with the presence of aromatic phenolic structures exhibiting strong π–π* transitions. The high absorbance intensity observed at these wavelengths further indicates a substantial concentration of UV‐active chromophores, confirming the chemical richness of the extract.

Variations in both absorption wavelength and intensity can be rationalized by substituent effects on the aromatic systems. Phenolic compounds bearing different functional groups are known to undergo bathochromic (red) and hyperchromic effects, depending on the nature and position of substituents. Bathochromic shifts are commonly associated with increased conjugation or higher degrees of hydroxylation and oxygenation, which stabilize excited electronic states. In flavonols, for instance, an increase in the number of hydroxyl groups typically induces a bathochromic shift of Band I by approximately 3–10 nm. Conversely, hyperchromic effects often arise from substitution phenomena such as methylation or glycosylation of hydroxyl groups in flavones and flavonols, leading to enhanced absorption intensity.

In the context of crude extracts, these spectral features may also reflect intermolecular interactions within the extract matrix, including hydrogen bonding or weak π–π interactions, which can further modulate the electronic transitions. Overall, the UV–visible spectral behavior provides complementary chemical evidence supporting the presence of structurally diverse phenolic constituents in *P. lentiscus*, in agreement with the LC‐MS profiling, and establishes a spectroscopic basis for interpreting the antioxidant and enzyme inhibitory activities discussed in subsequent sections.

### 3.2 LC‐MS Analysis of the Lactic Acid Extract Solution

The LC–MS analysis of the lactic acid extract enabled the unambiguous identification of several bioactive constituents based on their retention times (RT), molecular formulas, molecular weights, ionization modes, and characteristic ion transitions (Figure S1 and Table 1). The detected compounds reflect a chemically diverse profile dominated by phenolic acids and flavonoids, highlighting the effectiveness of lactic acid as a green extraction medium for recovering structurally heterogeneous bioactives.

**TABLE 1:** LC‐MS analysis of the lactic acid extract solution.

Number	*RT* (min)	Compounds	Molecular formula	Molecular weight (g/mol)	Ionization mode	Transition *m*/*z*
1	1.85	Catechol	C_6_H_6_O_2_	110.11	+	111 → 79
2	2.45	Caffeic acid	C_9_H_8_O_4_	180.16	—	179 → 135
3	2.65	Salicin	C_13_H_18_O_7_	286.28	+	287 → 269
4	2.95	Esculin	C_15_H_16_O_9_	340.28	—	339 → 295
5	3.25	Chlorogenic acid	C_16_H_18_O_9_	354.31	—	353 → 191
6	4.35	Catechin	C_15_H_14_O_6_	290.27	+	291 → 139
7	4.72	Epicatechin	C_15_H_14_O_6_	290.27	+	291 → 139
8	5.95	4‐Hydroxycoumarin	C_9_H_6_O_3_	162.14	+	161 → 117
9	6.40	Quercetin	C_15_H_10_O_7_	302.24	+	301 → 151
10	6.85	Verbascoside	C_29_H_36_O_15_	624.59	+	623 → 161
11	6.85	Myricetin	C_15_H_10_O_8_	318.24	+	317 → 179
12	7.15	Rutin	C_27_H_30_O_16_	610.52	+	609 → 301
13	7.85	Hesperidin	C_28_H_34_O_15_	610.56	+	609 → 303
14	8.92	Naringenin	C_15_H_12_O_5_	272.25	+	271 → 151
15	9.45	Silymarin	C_25_H_22_O_10_	482.44	+	481 → 125
16	9.85	Kaempferol	C_15_H_10_O_6_	286.24	+	285 → 133
17	10.12	Chrysin	C_15_H_10_O_4_	254.24	+	253 → 143
18	11.20	Cinnamic acid	C_9_H_8_O_2_	148.16	+	147 → 103

Notably, compounds such as myricetin, naringenin, epicatechin, chlorogenic acid, and quercetin were clearly identified through their specific chromatographic and mass spectrometric features. The coexistence of these molecules, which differ markedly in hydroxylation patterns, conjugation degrees, and molecular sizes, suggests a complex chemical matrix with a strong potential for redox‐related activity. The molecular weight distribution, ranging from low‐molecular‐weight phenolics (e.g., catechol) to more complex glycosylated structures such as rutin and verbascoside, further reflects the broad chemical spectrum of the extract.

The detection of compounds ionized in both positive (e.g., chrysin, kaempferol) and negative (e.g., caffeic acid, esculin) electrospray modes underlines their distinct ionization behaviors and functional group chemistries. This dual ionization pattern indicates differential interactions with the acidic extraction environment, which may influence compound stability and chemical reactivity. Such variability is particularly relevant when interpreting antioxidant performance, as ionization state and molecular structure are closely linked to electron‐donating capacity.

The detailed assignment of ion transitions provides a high level of confidence in compound identification and supports the robustness of the chemical profiling approach. This rigorous characterization is essential for establishing structure–activity relationships and for rationalizing the antioxidant and enzyme inhibitory activities observed experimentally. Moreover, the presence of well‐documented phenolics such as quercetin and silymarin reinforces the chemical relevance of the extract, as these compounds are widely recognized for their strong redox properties and interaction capabilities with biological targets. Overall, the results emphasize the chemical richness of the lactic acid extract and its suitability as a source of multifunctional phenolic compounds within a green chemistry framework.

### 3.3 Measurement of Bioactive Compounds of Lactic Acid Extract Solution

The green extraction of bioactive compounds from *P. lentiscus* using lactic acid enabled the efficient recovery of several classes of phytochemicals (Table 2). The lactic acid extract exhibited a remarkably high content of total phenolic compounds (142.13 ± 0.06 mg GAE/g DW) and flavonoids (16.17 ± 0.02 mg QE/g DW).

**TABLE 2:** Total polyphenol, flavonoid, and protein composition of the lactic acid extract solution.

Solution	Phenolic compounds (mg GAE/g DW)	Flavonoids (mg QE/g DW)
Lactic acid extract	142.13 ± 0.06	16.17 ± 0.02

The elevated phenolic content indicates that the combination of ultrasound‐assisted extraction and lactic acid constitutes an effective strategy for recovering polyphenolic metabolites from *P. lentiscus*. This efficiency may be attributed to the polar nature of lactic acid and its strong hydrogen‐bonding capacity, which facilitates interactions with hydroxylated phenolic structures and enhances mass transfer from the plant matrix. Similar observations have been reported in other plant systems where weak organic acids improved the solubilization of phenolic compounds by disrupting hydrogen‐bond networks within the plant cell wall matrix [6, 13].

The predominance of phenolic compounds in the extract is consistent with the well‐documented phytochemical profile of *P. lentiscus*, a species widely recognized as a rich source of tannins, flavonoids, and phenolic acids [5]. These secondary metabolites play important ecological and physiological roles in plants, including protection against oxidative stress, microbial pathogens, and herbivores. Their abundance in the obtained extract therefore reflects both the intrinsic metabolic composition of the species and the efficiency of the extraction system used [14].

Although present at lower concentrations than total phenolics, flavonoids were recovered in significant amounts. Flavonoids represent an important subclass of plant secondary metabolites characterized by diverse biological activities, including antioxidant and enzyme inhibitory properties. Their extraction under acidic conditions may be favored by improved stability and solubility of hydroxylated flavonoid structures at low pH [5, 13]. Furthermore, flavonoids can interact synergistically with other phenolic constituents, thereby collectively contributing to the biological properties of the extract.

From a phytochemical perspective, the high phenolic content observed in this study is comparable to, or even higher than, values previously reported for *P. lentiscus* extracts obtained using conventional solvents such as ethanol or methanol. For instance, Detti et al. [15] reported a phenolic content of 51.3 ± 1.8 mg GAE/g DW, while Azib et al. [16] recorded a value of 95.89 ± 1.88 mg GAE/g DW. Similarly, Garofulic et al. [17] reported values of 108.14 ± 2.12 mg GAE/g DW and 42.71 ± 0.93 mg GAE/g DW for leaf and fruit extracts, respectively. In addition, Yemmen et al. [18] reported phenolic levels of approximately 124.1 mg GAE/g DW in methanol–water extracts of *P. lentiscus* leaves collected in Tunisia, highlighting the high polyphenolic richness of this species.

Several studies have shown that acidic extraction media can enhance the release of bound phenolic compounds by partially disrupting interactions between these metabolites and structural macromolecules of the plant cell wall, such as polysaccharides and proteins. These mechanisms may partly explain the high recovery of phenolic compounds observed in the present study.

In contrast, the flavonoid content obtained in this study falls within the range reported for several *P. lentiscus* extracts. Previous studies have reported flavonoid concentrations ranging from 4 mg to 13 mg QE/g DW depending on the extraction solvent and plant part analyzed. Such variability is generally attributed to differences in extraction techniques, plant origin, environmental conditions, and metabolic variability within the species.

For example, Zaidi et al. [5] reported a flavonoid content of 13.24 ± 1.59 mg QE/g DW, which is comparable to the value obtained in the present study. Lower concentrations were reported by Dahmoune et al. [19] using microwave‐assisted, ultrasound‐assisted, and accelerated solvent extraction methods, with values of 5.16 ± 0.22, 4.61 ± 0.02, and 4.79 ± 0.03 mg QE/g DW, respectively. Similarly, Azib et al. [16] reported a value of 5.18 ± 0.05 mg QE/g DW using conventional extraction methods. The relatively higher flavonoid content obtained in the present study therefore suggests that the lactic acid‐based extraction system may enhance the recovery of these metabolites from *P. lentiscus*.

### 3.4 Antioxidant Activity

The strong antioxidant activity observed in the lactic acid extract of *P. lentiscus* further confirms the presence of a high concentration of redox‐active metabolites (Table 3). The DPPH radical‐scavenging activity (189.69 ± 4.57 mmol TE/100 g DW) indicates a pronounced capacity of the extract to neutralize free radicals through hydrogen atom transfer or single‐electron transfer mechanisms. Such activity is typically associated with phenolic compounds containing multiple hydroxyl groups capable of stabilizing radical species. Notably, the free‐radical scavenging capacity of *P. lentiscus* extract exceeds that reported for clove, oregano, and pomegranate extracts, which showed values of 64.4 ± 3.70, 35.5 ± 1.15, and 141 ± 12.6 mmol TE/100 g DW, respectively [20].

**TABLE 3:** Antioxidant potential of *Pistacia lentiscus* crude extract.

Type of assay	Antioxidant potential
DPPH•	189.69 ± 4.57 mmol TE/100 g DW
Reducing power	282.27 ± 3.79 mg AAE/g DW

Similarly, the reducing power assay revealed a substantial electron‐donating capacity (282.27 ± 3.79 mg AAE/g DW), reflecting the extract's ability to reduce ferric ions (Fe^3^^+^) to ferrous ions (Fe^2^^+^). The agreement between DPPH scavenging and reducing power results suggests a coherent antioxidant profile, where the extracted metabolites act both as direct radical scavengers and as reducing agents capable of mitigating oxidative processes. These observations are consistent with previous reports showing a positive correlation between total phenolic content and antioxidant activity in plant extracts.

The reducing power observed in this study is markedly higher than values reported for several commonly consumed plant‐derived foods and herbs. For instance, rosemary exhibited a reducing capacity of 84.5 mg ascorbic acid/g DW, followed by thyme (65.9 mg/g DW), oregano (65.9 mg/g DW), and basil (65.1 mg/g DW). Other plant materials such as turmeric powder (56.8 mg/g DW), walnut flour (55.6 mg/g DW), and yarrow (49.4 mg/g DW) showed lower reducing capacities, while beetroot powder (40.9 mg/g DW), spinach powder (25.8 mg/g DW), tomato powder (18.9 mg/g DW), chili pepper (15.1 mg/g DW), and red pepper (21.4 mg/g DW) were considerably less active. The lowest activities were detected in brewer's yeast flakes (11.9 mg/g DW) and dried onion (11.0 mg/g DW). In comparison, the reducing power of the *P. lentiscus* extract in the present study was approximately 3–5 times higher than that of the most active species reported, including rosemary. This pronounced difference highlights the particularly strong electron‐donating capacity of the extract, typically associated with a high concentration of polyphenols and flavonoids [21].

The elevated reducing power can also be attributed to the efficiency of the lactic acid‐assisted extraction system, which likely facilitated the release of phenolic compounds bound to the plant matrix. Since reducing power reflects the ability of antioxidants to donate electrons and convert ferric (Fe^3^^+^) to ferrous (Fe^2^^+^) ions, the high values obtained further confirm the strong antioxidant potential of *P. lentiscus* extracts.

Moreover, the observed antioxidant activity may be influenced by the extraction conditions. The acidic environment provided by lactic acid can promote the liberation of matrix‐bound phenolic compounds while limiting oxidative degradation during extraction. This protective effect helps preserve both the structural integrity and the redox activity of the extracted metabolites, resulting in an extract with a robust and stable antioxidant profile.

### 3.5 Antienzymatic Activities

In this study, the impact of lactic acid extract was evaluated through various enzymatic activities, particularly the inhibition of acetyl cholinesterase and urease (Table 4).

**TABLE 4:** Enzyme activity of lactic acid extract solution.

IC_50_ (%)	Acetylcholine esterase inhibition	Urease inhibition
Lactic acid extract	0.29	1.49
Standard	—	1.50

The inhibitory effects of the lactic acid extract of P. lentiscus on acetylcholinesterase were evaluated at concentrations ranging from 0.05% to 1.5%. The extract exhibited a pronounced concentration‐dependent inhibition, increasing from 29.40% to 62.28%, with an IC_50_ of 0.29%, indicating a strong inhibitory potential. This activity can be attributed to the presence of bioactive compounds, notably phenolic acids, flavonoids, and tannins, which likely enhance the effect. Notably, no activity was observed for the reference compound used (gallic acid), highlighting the significant bioactive potential of the lactic acid extract of P. lentiscus.

AChE is a key enzyme involved in both cholinergic and non‐cholinergic pathways, and its inhibition is a widely recognized target for modulating neurological processes [22]. The observed inhibitory effect highlights the capacity of plant‐derived phenolics to act as potent modulators of AChE activity. Among the identified compounds, caffeic acid, chlorogenic acid, kaempferol, quercetin, and myricetin have been reported to inhibit AChE effectively, with phenolic acids active around 2 mmol/L and flavonoids between 0.63 mmol/L and 0.7 mmol/L [23]. Compared to other plant species, such as *Globularia alypum*, *Scilla maritima*, *Eryngium maritimum*, *Marrubium vulgare*, and *Myrtus communis* [24], the lactic acid extract of *P. lentiscus* displayed higher inhibitory activity, reflecting its rich chemical composition.

The inhibition is likely mediated by multiple structural features of the molecules, including the number and position of hydroxyl groups, oxygen atoms, and hydrophobic moieties, which facilitate hydrogen bonding and π–π interactions with the enzyme active site. Flavonoids such as chrysin and hesperidin, also detected in the extract, exhibited potent inhibitory activity with IC_50_ values of 31.61 and 0.85 nM, respectively [25], supporting the contribution of both aglycone and glycosylated structures to enzyme binding.

Similarly, the urease inhibitory activity of the extract was evaluated at concentrations ranging from 0.2% to 1.5%, yielding inhibition rates between 4.63% and 51.29%, with an IC_50_ value of 1.49%. This value is significantly comparable to that recorded for the reference compound (IC_50_ = 1.50%), indicating a stronger inhibitory potential of the extract. This confirms that lactic acid extraction effectively concentrates bioactive compounds capable of targeting urease, an enzyme critical for the survival of pathogens such as *Helicobacter pylori* [1]. The inhibitory effect is primarily attributed to phenolic acids and flavonoids, including gallic acid, hesperidin, quercetin, rutin, myricetin, chrysin, naringenin, and kaempferol, which interact with urease through hydrogen bonding and van der Waals forces [26], stabilizing the enzyme–ligand complex.

Overall, these results emphasize the structural diversity of *P. lentiscus* phenolics and flavonoids as a major factor in the synergistic inhibition of both AChE and urease. The combination of hydroxyl‐rich moieties, conjugated systems, and glycosylation patterns appears to modulate binding affinity and inhibitory efficiency. This study demonstrates that lactic acid represents a green and effective extraction medium for enriching bioactive compounds with multifunctional enzyme modulatory potential, providing a basis for further mechanistic investigations and the development of natural inhibitors.

### 3.6 Molecular Docking

Molecular docking simulations are an essential tool for exploring the active site of a protein and elucidating the interactions between ligands and biological molecules. In the present study, the compounds characterized in the *P. lentiscus* leaf extract were selected to investigate their binding affinity and interactions with the acetylcholinesterase and urease enzymes (Figure 2).

**FIGURE 2:**
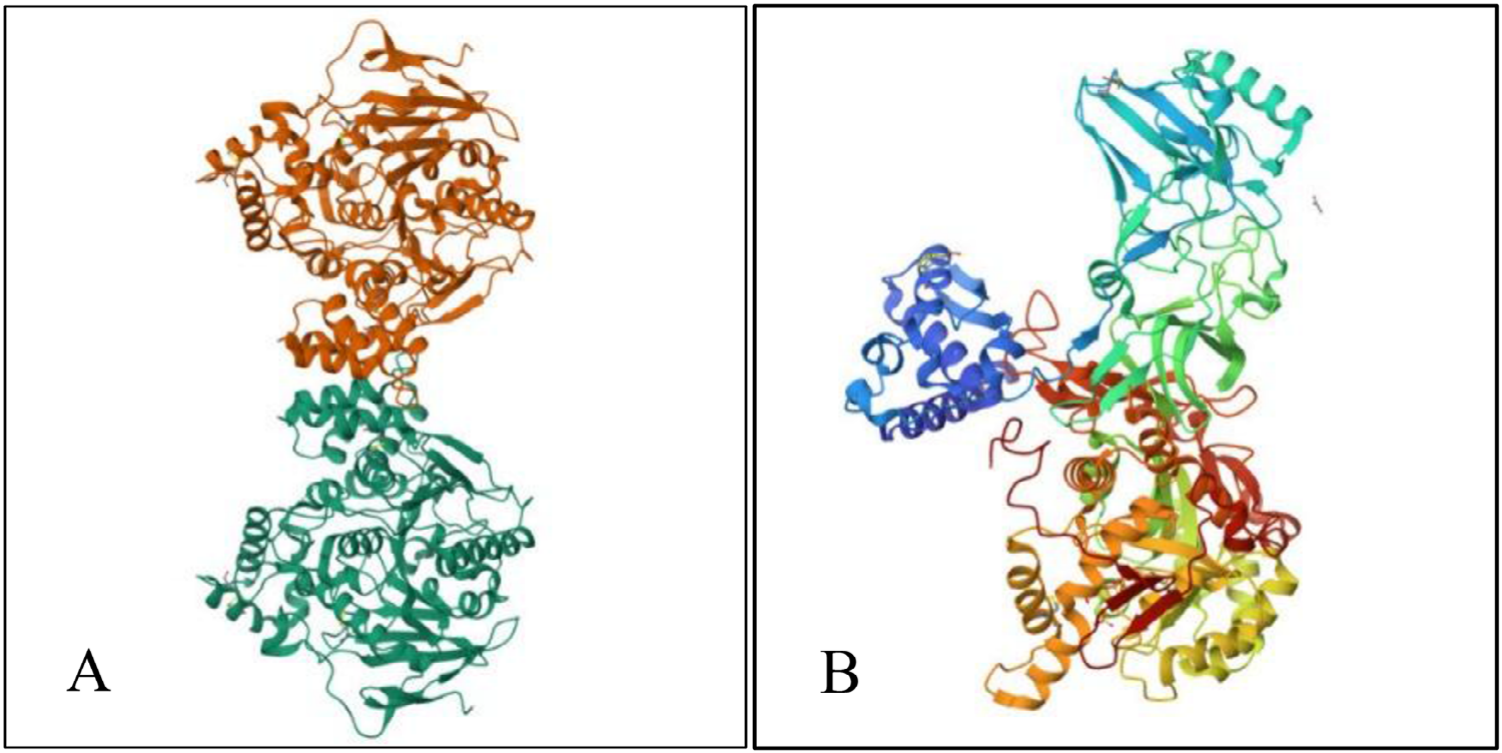
Structural representation of acetylcholinesterase (A) and urease (B) enzymes.

The results of molecular docking performed on phenolic compounds against acetylcholinesterase show a wide variability in binding energies and interaction modes (Table 5). The molecular docking of hesperidin showed an affinity of −10.2 kcal/mol and an inhibition constant of 7.50 µM, primarily attributable to interactions with residues TRP A:286, TYR A:341, and TYR A:124 through π–π stacked and π–π T‐shaped interactions, as well as a π–sigma interaction with residues TYR A:337 and TRP A:86. Additionally, interactions with HIS A:287 and GLY A:121 were observed via π–alkyl and carbon–hydrogen bonds, respectively.

**TABLE 5:** Docking results and protein–ligand interaction scores of *Pistacia lentiscus* extract compounds with acetylcholinesterase.

Compounds	Molecular formula	Binding energy (kcal/mol)	Predicted inhibitory constant 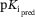 (µM)	Interacting residues	No. of H bonds
Chlorogenic acid	C_16_H_18_O_9_	−8.8	6.47	TRP A:286 TYR A:341 SER A:125	2
Caffeic acid	C_9_H_8_O_4_	−7.1	5.22	TYR A:337 TRP A:86 GLU A:202 GLY A:448	2
Cinnamic acid	C_9_H_8_O_2_	−6.7	4.93	TYR A:337 TRP A:86	1
Catechol	C_6_H_6_O_2_	−5.2	3.83	TRR A:337 TRP A:86	0
Myricetin	C_15_H_10_O_8_	−9.8	7.21	TYR A:337 TYR A:72 ASN A:87 TYR A:124 SER A:125 TRP A:86	4
Quercetin	C_15_H_10_O_7_	−8.6	6.33	SER A:293 ARG A:296 TYR A:124 TYR A:337 TRP A:286 TYR A:341	4
Kaempferol	C_15_H_10_O_6_	−9.3	6.84	ASN A:87 ASP A:74 SER A:125 TRP A:86 TYR A:124	1
Naringenin	C_15_H_12_O_5_	−8.8	6.47	TYR A:124 TRP A:286 TYR A:341 TYR A:337	1
Hesperidin	C_28_H_34_O_15_	−10.2	7.50	HIS A:287 TRp A:286 TYR A:337 TRP A:86 TYR A:341 TYR A:124 GLY A:121	0
Epicatechin	C_15_H_14_O_6_	−8.2	6.03	TYR A:124 TYR A:337 PHE A:295 TYR A:341 TYR A:72 TRP A:286	5
Catechin	C_15_H_14_O_6_	−9.5	6.99	HIS A:447 TYR A:449 TRP A:439 TRP A:86 ASP A:74 THR A:83 TYR A:124 TYR A:337	5
Chrysin	C_15_H_10_O_4_	−9.1	6.69	PHE A:295 TYR A:124 TRP A:286 TYR A:341	2
Rutin	C_27_H_30_O_16_	−7.5	5.51	TRP A:86 GLN A:71 TYR A:341 SER A:293 ARG A:296 TYR A:337 PHE A:295 ASP A:74 LEU A:76 TRP A: 286 ASN A: 87 SER A:125 TYR A:124	6
Verbascoside	C_29_H_36_O_15_	−10.1	7.43	TYR A:341 ASP A:74 THR A:83 TYR A:124 SER A:125 SER A:203 GLY A:120 TRP A:286 TRP A:86 GLY A:121	7
Silymarin	C_25_H_22_O_10_	−9.3	6.84	SER A:293 PHE A:295 ARG A:296 TYR A:337 THR A:83 TRP A:86 TRP A:439 TYR A:449 TRP A:286 TYR A:124 Val:294	5
Aesculin	C_15_H_16_O_9_	−8.6	6.33	ASN A:87 TYR A:124 TYR A:337 ASP A:74 TYR A:341 TRP A:286 THR A:83	5
4‐Hydroxycoumarin	C_9_H_6_O_3_	−7.1	5.22	TRP A:286	0
Salicin	C_13_H_18_O_7_	−7.8	5.74	TYR A:337 SER A:125 TYR A:124 THR A:83 GLY A:82 TRP A:86 HIS A:447	4

The predicted binding model for verbascoside revealed an affinity energy of −10.1 kcal/mol and an inhibition constant of 7.43 µM, established through hydrogen bonds with residues TYR A:341, ASP A:74, THR A:83, TYR A:124, SER A:125, and SER A:203, as well as π–π stacked and π–π T‐shaped interactions with residues GLY A:120 and TRP A:286. Additional interactions, including π–donor hydrogen bonds and van der Waals forces, involved TRP A:86 and GLY A:121, respectively.

The interaction of myricetin within the catalytic cavity displayed an affinity of −9.8 kcal/mol and an inhibition constant of 7.21 µM, mainly explained by four hydrogen bonds involving residues TYR A:337, TYR A:72, and ASN A:87. π–π stacked and π–π T‐shaped interactions with TYR A:124 and TRP A:86 were also observed, along with a π–donor hydrogen bond with SER A:125.

Docking of catechin at the enzyme's active site revealed strong interactions, particularly with HIS A:447, TYR A:449, TRP A:439, TRP A:86, and ASP A:74 through hydrogen bonds, as well as carbon–hydrogen and π–donor hydrogen interactions with TYR A:124. π–π stacked and π–π T‐shaped interactions involving TYR A:337 and TRP A:86 were also established, in addition to an unfavorable donor–donor interaction with THR A:83. Overall, this corresponded to an affinity of −9.5 kcal/mol and an inhibition constant of 6.99 µM.

Docking of kaempferol also showed a high affinity for active site residues, with an energy of −9.3 kcal/mol and an inhibition constant of 6.84 µM. This affinity is attributed to hydrogen bonds with ASN A:87, stabilized by π–π stacked and π–π T‐shaped interactions involving TRP A:86 and TYR A:124, as well as π–donor hydrogen bonds and an unfavorable donor–donor interaction with SER A:125 and ASP A:74, respectively.

Silymarin primarily formed five hydrogen bonds with SER A:293, PHE A:295, ARG A:296, TYR A:337, and THR A:83. π–π Stacked and π–π T‐shaped interactions were also observed with TRP A:86 and TRP A:286, along with π–alkyl interactions with TRP A:439 and TYR A:449. π–lone pair and carbon–hydrogen bonds with TYR A:124 and VAL A:294 were also formed, resulting in an affinity of −9.3 kcal/mol and an inhibition constant of 6.84 µM.

Docking simulation of chrysin showed an affinity of −9.1 kcal/mol and an inhibition constant of 6.69 µM, mediated by hydrogen bonds with PHE A:295 and TYR A:124, along with π–π T‐shaped interactions with TRP A:286 and TYR A:341.

The predicted model for chlorogenic acid revealed hydrogen bonds with SER A:125 and TYR A:341, as well as π–π stacked interactions with TRP A:286 and TYR A:341, corresponding to an affinity of −8.8 kcal/mol and an inhibition constant of 6.47 µM. Docking of naringenin revealed an affinity of −8.8 kcal/mol and an inhibition constant of 6.47 µM, associated with hydrogen bonds to TYR A:124 and π–π stacked interactions with TRP A:286 and TYR A:341. Additionally, an unfavorable donor–donor interaction with TYR A:337 was observed.

The predicted quercetin interaction showed an affinity of −8.6 kcal/mol and an inhibition constant of 6.33 µM, mediated by hydrogen bonds with SER A:293, ARG A:296, TYR A:124, and TYR A:337, as well as π–π stacked interactions with TRP A:286 and TYR A:341.

Docking simulation of esculin revealed an affinity of −8.6 kcal/mol and an inhibition constant of 6.33 µM, stabilized by hydrogen bonds with ASN A:87, TYR A:124, and TYR A:337, along with π–π stacked and π–π T‐shaped interactions with TYR A:341 and TRP A:286. Carbon–hydrogen bonding with ASP A:74 and an unfavorable donor–donor interaction with THR A:83 were also detected.

The predicted model for epicatechin showed an affinity of −8.2 kcal/mol and a predicted inhibition constant of 6.03 µM. This model was distinguished by hydrogen bonds with TYR A:124, TYR A:337, PHE A:295, TYR A:341, and TYR A:72, as well as a π–π stacked aromatic interaction with TRP A:286, suggesting stable anchoring within the catalytic gorge.

Salicin, with an affinity of −7.8 kcal/mol and an inhibition constant of 5.74 µM, formed multiple hydrogen bonds (TYR A:337, SER A:125, TYR A:124, THR A:83), complemented by a π–π stacked interaction with TRP A:86 and carbon–hydrogen bonds with GLY A:82 and HIS A:447, reinforcing its anchoring within the catalytic gorge.

The molecular docking of rutin showed an affinity of −7.5 kcal/mol and an inhibition constant of 5.51 µM, with multiple hydrogen bonds (TRP A:86, GLN A:71, TYR A:341, SER A:293, ARG A:296, TYR A:337) as well as π–π T‐shaped, π–alkyl, and π–anion interactions with TRP A:286, LEU A:76, and ASP A:74, respectively. Carbon–hydrogen and π–donor hydrogen bonds involving ASN A:87, SER A:125, and TYR A:124 were also observed, along with an unfavorable donor–donor interaction with PHE A:295.

Caffeic acid exhibited an affinity of −7.1 kcal/mol and an inhibition constant of 5.22 µM, involving hydrogen bonds with TYR A:337 and GLU A:202, π–π stacked interactions with TRP A:86, and a carbon–hydrogen bond with GLY A:448, indicating a correct but less stable interaction than that of flavonoids.

4‐Hydroxycoumarin displayed an affinity of −7.1 kcal/mol and an inhibition constant of 5.22 µM, relying exclusively on a π–π stacked interaction with TRP A:286.

Regarding cinnamic acid, an affinity of −6.7 kcal/mol and an inhibition constant of 4.93 µM was determined, supported by a hydrogen bond with TYR A:337 and a π–π stacked interaction with TRP A:86, indicating weaker anchoring.

Finally, catechol exhibited the lowest affinity (−5.2 kcal/mol, Ki 3.83 µM), with only two π–π stacked interactions involving TRP A:337 and TRP A:86, suggesting limited interaction due to its simple and minimally substituted structure.

The molecular docking results suggest that phenolic compounds from *P. lentiscus* inhibit acetylcholinesterase through a multifactorial mechanism involving both occupation of the catalytic gorge and interactions with key residues of the active site as well as the peripheral anionic site (PAS). In particular, π–π stacking and T‐shaped π–π interactions observed with aromatic residues such as TRP A:86, TRP A:286, TYR A:124, TYR A:337, and TYR A:341 indicate a strong affinity of phenolic cores for the hydrophobic regions of the catalytic channel, promoting stable ligand anchoring. Simultaneously, the formation of hydrogen bonds with essential residues including SER A:203 (a member of the catalytic triad), HIS A:447, ASP A:74, SER A:125, and ASN A:87 suggests a direct disruption of the catalytic hydrolysis mechanism of acetylcholine.

Complex flavonoids and polyphenols such as hesperidin, verbascoside, myricetin, catechin, and quercetin therefore exhibit a dual mode of action: (i) competitive inhibition through occupation of the active site, preventing substrate access, and (ii) non‐competitive or mixed inhibition via interactions with the PAS (notably TRP A:286 and TYR A:341), which may induce conformational changes that reduce enzymatic efficiency. In contrast, simple phenolic compounds such as caffeic acid, cinnamic acid, and catechol display lower affinity due to a limited number of functional groups capable of forming stabilizing interactions. These findings confirm that inhibitory efficiency is strongly correlated with structural richness in aromatic rings and hydroxyl groups, which determine the multiplicity of interactions and the stability of the enzyme–ligand complex.

The molecular docking results further support these observations. All investigated compounds exhibit significant affinity toward the catalytic cavity of AChE, consistent with the well‐established interaction propensity of polyphenolic structures. Large and highly hydroxylated molecules such as hesperidin, verbascoside, myricetin, and silymarin display the most favorable binding energies (−10.2 to −9.3 kcal/mol). This enhanced affinity can be attributed to their structural complexity, which enables the simultaneous formation of multiple hydrogen bonds and aromatic interactions with key active‐site residues, including TRP A:286, TYR A:124, TYR A:337, and TYR A:341. These interaction patterns are consistent with previous reports demonstrating that a high density of hydroxyl groups combined with extended conjugated systems significantly improves anchoring within enzyme catalytic sites [27].

Flavonoids of intermediate structural complexity, including catechin, chrysin, quercetin, esculin, and naringenin, also exhibit strong binding affinities (−9.0 to −8.6 kcal/mol). Their docking poses reveal the formation of hydrogen bond networks involving residues such as ASN A:87, SER A:125, and TYR A:124, together with aromatic stacking interactions with TRP A:286 or TYR A:341. These observations are in good agreement with literature data highlighting the essential role of hydrogen bonding and aromatic interactions in flavonoid‐mediated enzyme binding and inhibition [28, 29]. Although occasional unfavorable donor–donor interactions were detected for some ligands, these did not substantially compromise complex stability, indicating that overall molecular geometry, rigidity, and conjugation play a decisive role in maintaining high affinity [2].

In contrast, structurally simple phenolics such as caffeic acid, cinnamic acid, 4‐hydroxycoumarin, and catechol display comparatively lower binding energies (−7.1 to −5.2 kcal/mol). Their interaction modes are generally limited to a small number of hydrogen bonds or isolated π–π stacking interactions, reflecting a reduced capacity to engage the active site through multiple non‐covalent contacts. This behavior aligns with earlier findings showing that simple phenolics exhibit more restricted inhibitory potential due to their lower density of interaction sites and limited aromatic surface area [30].

Collectively, these results emphasize a clear structure–affinity relationship, in which the multiplicity and diversity of non‐covalent interactions—particularly those involving aromatic residues—govern the stability and strength of ligand–enzyme complexes. Within this framework, highly hydroxylated and/or glycosylated flavonoids emerge as the most promising AChE inhibitors, in full agreement with previous studies reporting their strong capacity to modulate enzymatic activity through multivalent interaction mechanisms [31].

Regarding molecular docking against urease (Table 6), hesperidin exhibits a strong affinity for the enzyme, characterized by a binding energy of –9.6 kcal/mol and a predicted inhibition constant of 7.06 µM. Its binding mode involves multiple types of interactions, including hydrogen bonds with residues GLU A:9, ASP A:730, SER A:421, GLU A:418, and LYS A:716. Additionally, π–sigma and carbon–hydrogen interactions are formed with THR A:33 and VAL A:744, respectively, along with π–alkyl interactions with PRO A:743 and PHE A:838. Electrostatic interactions, such as π–cation and π–anion, are also observed with LYS A:745 and GLU A:742.

**TABLE 6:** Docking results and protein–ligand interaction scores of *Pistacia lentiscus* extract compounds with urease.

Compounds	Molecular formula	Binding energy (kcal/mol)	Predicted inhibitory constant 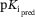 (µM)	Interacting residues	No. of H bonds
Chlorogenic acid	C_16_H_18_O_9_	−8.0	5.89	GLU A:718 LYS A:709 GLU A:742 THR A:33 LYS A:716 ASP A:730 PHE A:712 VAL A:744	4
Caffeic acid	C_9_H_8_O_4_	−6.2	4.56	THR A:829 THR A:578 ASN A:580 GLN A:649 ARG A:646 VAL A:831	5
Cinnamic acid	C_9_H_8_O_2_	−5.4	3.97	TYR A:32 PHE A:712 LYS A:716 ASP A:730	1
Catechol	C_6_H_6_O_2_	−4.8	3.53	LYS A:716 PHE A:712 ASP A:730	1
Myricetin	C_15_H_10_O_8_	−7.8	5.74	GLU A:718 GLN A:82 GLU A:742 ASP A:730 VAL A:744 THR A:740 LYS A:716 PHE A:712 TYR A:32 VAL A:36	3
Quercetin	C_15_H_10_O_7_	−8.3	6.11	GLN A:82 ALA A:80 GLU A:742 LYS A:716 THR A:740 VAL A:744 TYR A:32 ASP A:730 GLU A:718 PHE A:712	4
Kaempferol	C_15_H_10_O_6_	−8.0	5.89	LYS A:716 GLU A:718 GLN A:82 GLU A:742 ASP A:730 THR A:740 PHE A:712 VAL A:744 TYR A:32	4
Naringenin	C_15_H_12_O_5_	−7.9	5.81	THR A:33 GLU A:718 GLU A:742 VAL A:36 PHE A:712 LYS A:716 ASP A:730	2
Hesperidin	C_28_H_34_O_15_	−9.6	7.06	GLU A:9 ASP A:730 SER A:421 GLU A:418 LYS A:716 LYS A:745 PRO A:743 GLU A:742 THR A:33 VAL A:744 PHE A:838	6
Epicatechin	C_15_H_14_O_6_	−8.1	5.96	THR A:33 VAL A:744 GLU A:718 LYS A:709 LYS A:716 ASP A:730 PHE A:712	4
Catechin	C_15_H_14_O_6_	−8.1	5.96	LYS A:709 LYS A:716 ALA A:37 PHE A:712 ASP A:730 THR A: 33 VAL A: 36 GLU A: 742	3
Chrysin	C_15_H_10_O_4_	−7.9	5.81	GLU A:742 THR A:33 ASP A:730 PHE A:712 LYS A:716	1
Rutin	C_27_H_30_O_16_	−9.0	6.62	ASP A:730 GLU A:742 SER A:421 GLU A:418 LEU A:838 ALA A:16 PHE A:838 LYS A:716	5
Verbascoside	C_29_H_36_O_15_	−9.2	6.77	LYS A:709 TYR A:32 MET A:746 LYS A:716 GLU A:418 VAL A:744 GLU A:742 PHE A:712 ASP A:730 ALA A:16 ALA A:37	7
Silymarin	C_25_H_22_O_10_	−9.0	6.62	GLU A:418 SER A:421 LYS A:709 VAL A:744 LYS A: 716 LYS A: 745 TRP A: 728 ASP A: 730 GLU A: 718	4
Aesculin	C_15_H_16_O_9_	−8.1	5.96	ALA A:80 THR A:740 LYS A:716 LYS A:709 GLU A:718 VAL A:744 ASP A:730 TYR A:32	5
4‐Hydroxycoumarin	C_9_H_6_O_3_	−6.3	4.63	LYS A:716 LYS A:709 PHE A:712 ASP A:730 VAL A:744	2
Salicin	C_13_H_18_O_7_	−6.5	4.78	GLU A:649 THR:578 SER A:834 GLU A:642 PHE A:838 LEU A:833	4

The interaction mode of verbascoside with the enzyme shows a binding energy of –9.2 kcal/mol and an inhibition constant of 6.77 µM. Its anchoring is mediated by several hydrogen bonds (LYS A:709, TYR A:32, MET A:746, LYS A:716, GLU A:418, VAL A:744, GLU A:742) and π–alkyl interactions with ALA A:16, ALA A:37, and PHE A:838. It also forms a π–π stacked interaction with PHE A:712 and a π–anion interaction with ASP A:730.

Docking simulation of silymarin shows the same binding energy as verbascoside (–9.2 kcal/mol; Ki 6.77 µM), but with a distinct anchoring mode: hydrogen bonds with GLU A:418, SER A:421, LYS A:709, VAL A:744; T‐shaped π–π interactions with TRP A:728; π–sigma interactions with LYS A:745; and carbon–hydrogen interactions with GLU A:718. Additionally, π–anion and π–cation interactions are observed with LYS A:716 and ASP A:730.

The docking of rutin shows a binding energy of –9.0 kcal/mol and an inhibition constant of 6.62 µM. Its interactions include hydrogen bonds with ASP A:730, GLU A:742, SER A:421, and GLU A:418, along with π–alkyl interactions involving LEU A:838, ALA A:16, PHE A:838, and LYS A:716.

The predicted binding mode of quercetin presents a binding energy of –8.3 kcal/mol and a Ki of 6.11 µM. It interacts with the enzyme via multiple hydrogen bonds (GLN A:82, ALA A:80, GLU A:742, LYS A:716) and forms π–π stacked and T‐shaped interactions with TYR A:32 and PHE A:712. π–Sigma, unfavorable acceptor–acceptor, and π–anion interactions are also observed with VAL A:744, GLU A:718, and ASP A:730, respectively. Additionally, π–donor hydrogen bonds and carbon–hydrogen interactions are formed with THR A:740 and PHE A:712.

Predictive docking of epicatechin, catechin, and esculin shows identical affinities (–8.1 kcal/mol; Ki 5.96 µM), indicating strong inhibitory potential. Epicatechin forms four hydrogen bonds (THR A:33, VAL A:744, GLU A:718, LYS A:709), a π–π stacking interaction with PHE A:712, and π–alkyl and π–anion interactions with LYS A:716 and ASP A:730, respectively. Catechin forms three hydrogen bonds with LYS A:709, LYS A:716, and ALA A:37, a π–π stacked interaction with PHE A:712, and additional hydrophobic interactions (π–alkyl and π–sigma) with VAL A:36 and THR A:33, along with π–anion interactions with ASP A:730 and GLU A:742. Esculin establishes multiple hydrogen bonds with ALA A:80, THR A:740, LYS A:716, LYS A:709, and GLU A:718, a T‐shaped π–π interaction with TYR A:32, and π–alkyl and π–anion interactions with VAL A:744 and ASP A:730, respectively.

Predictive docking of kaempferol and chlorogenic acid shows similar binding energies (–8.0 kcal/mol; Ki 5.89 µM). Kaempferol forms four hydrogen bonds with LYS A:716, GLU A:718, GLN A:82, and GLU A:742, along with π–π stacked and T‐shaped interactions with PHE A:712 and TYR A:32, and π–sigma, carbon–hydrogen, and π–anion interactions with VAL A:744, THR A:740, and ASP A:730, respectively. Chlorogenic acid also forms four hydrogen bonds with GLU A:718, LYS A:709, GLU A:742, and THR A:33, supported by π–π stacked, π–alkyl, carbon–hydrogen, and π–anion interactions with PHE A:712, LYS A:716, VAL A:744, and ASP A:730, respectively.

The proposed anchoring modes of naringenin and chrysin show identical binding energies (–7.9 kcal/mol; Ki 5.81 µM). Naringenin forms two hydrogen bonds with THR A:33 and GLU A:718, π–π stacked interactions with PHE A:712, π–alkyl interactions with VAL A:36 and LYS A:716, and π–anion interactions with GLU A:742 and ASP A:730. In contrast, chrysin forms only one hydrogen bond with GLU A:742 but establishes π–π stacked, π–sigma, π–alkyl, and π–anion interactions with PHE A:712, THR A:33, LYS A:716, and ASP A:730, respectively.

Docking of myricetin shows a binding energy of –7.8 kcal/mol and an inhibition constant of 5.74 µM. Its interaction profile includes hydrogen bonds with GLU A:718, GLN A:82, and GLU A:742, π–π stacked and T‐shaped interactions with PHE A:712 and TYR A:32, as well as π–alkyl, carbon–hydrogen, unfavorable donor–donor, and π–anion interactions with LYS A:716, VAL A:36, THR A:740, VAL A:744, and ASP A:730, respectively.

The modeled interaction of salicin exhibits a binding energy of –6.5 kcal/mol and an inhibition constant of 4.78 µM, mediated by hydrogen bonds with GLU A:649 and THR A:578, carbon–hydrogen interactions with LEU A:833 and SER A:834, a π–π stacked interaction with PHE A:838, and a π–anion interaction with GLU A:642.

The predicted model of 4‐hydroxycoumarin shows a binding energy of –6.3 kcal/mol and a Ki of 4.63 µM. This model is characterized by two hydrogen bonds with LYS A:716 and LYS A:709, a π–π stacked interaction with PHE A:712, and π–alkyl and π–anion interactions with VAL A:744 and ASP A:730, respectively.

The proposed binding configuration for caffeic acid has a binding energy of –6.2 kcal/mol and a Ki of 4.56 µM. This molecule forms five hydrogen bonds with THR A:829, THR A:578, ASN A:580, and GLN A:649, a π–alkyl interaction with VAL A:831, and two π–cation electrostatic interactions with ARG A:646.

Simulated docking of cinnamic acid shows intermediate affinity (–5.4 kcal/mol; 3.97 µM), forming a single hydrogen bond with TYR A:32, a π–π stacked interaction with PHE A:712, a π–alkyl interaction with LYS A:716, and a π–anion interaction with ASP A:730.

Finally, catechol exhibits the lowest affinity (–4.8 kcal/mol; 3.53 µM), forming a single hydrogen bond with LYS A:716, a π–π stacked interaction with PHE A:712, a π–alkyl interaction with LYS A:716, and a π–anion interaction with ASP A:730.

The molecular docking results demonstrate that all investigated compounds exhibit notable affinity toward the catalytic cavity of urease, in line with the well‐documented interaction propensity of phenolic compounds. The binding modes reveal recurrent interactions with catalytically relevant acidic residues, notably Glu A:418, Glu A:718, Glu A:742, Glu A:649, and Asp A:730, which are known to play a central role in ligand recognition, stabilization, and enzymatic regulation within the urease active site. These residues, located along the access channel and within the catalytic pocket, actively participate in substrate orientation and inhibitor anchoring. In particular, Glu A:418 and Glu A:649 appear to be involved in the early recognition and guidance of ligands toward the catalytic cavity, whereas Glu A:718 and Glu A:742 contribute to complex stabilization through hydrogen bonding and electrostatic interactions. Asp A:730 emerges as a key inhibitory hotspot, forming hydrogen bonds and π–anion or electrostatic interactions with a large number of ligands, thereby enhancing their anchoring within the catalytic cavity. Collectively, these acidic residues are essential for maintaining the active‐site architecture and modulating enzymatic activity, making them critical targets for urease inhibitor design [32, 33, 34].

Within this framework, hesperidin, rutin, silymarin, and verbascoside appear as the most relevant ligands, as they simultaneously interact with multiple key catalytic residues and are stabilized by dense hydrogen‐bonding networks. The predicted inhibition mechanism likely involves a combination of Ni^2^^+^ coordination perturbation, stabilization of reaction intermediates, and electrostatic interactions with charged active‐site residues, ultimately contributing to the disruption of urease catalytic function. Such multivalent binding behavior is characteristic of large, highly functionalized polyphenols and is consistent with their superior docking scores.

In addition to acidic residues, several tested compounds—including chlorogenic acid, cinnamic acid, catechol, myricetin, quercetin, kaempferol, naringenin, hesperidin, epicatechin, catechin, chrysin, rutin, verbascoside, silymarin, esculin, and 4‐hydroxycoumarin—interact with basic residues located near the access channel, such as LYS A:716, LYS A:709, and LYS A:745, while caffeic acid additionally interacts with ARG A:646. These interactions suggest a possible blockage of substrate entry into the catalytic cavity by interfering with electrostatic guidance mechanisms required for urea stabilization. In particular, interaction with ARG A:646 may disrupt the recognition of charged ligands and contribute to inhibition by destabilizing substrate–enzyme electrostatic complementarity [35]. Among the studied compounds, silymarin stands out by interacting with three key basic residues, whereas most other ligands interact with one or two, highlighting its strong anchoring capacity.

Hydrophobic and aromatic residues also play a decisive role in ligand stabilization. Hesperidin, rutin, and salicin interact with PHE A:838, while silymarin exhibits a specific interaction with TRP A:728. Other compounds, including cinnamic acid, myricetin, quercetin, kaempferol, verbascoside, and esculin, adopt a multiple‐interaction binding mode involving TYR A:32 and PHE A:712, whereas chlorogenic acid, catechol, epicatechin, catechin, chrysin, and 4‐hydroxycoumarin interact predominantly with PHE A:712. These π–π stacking and hydrophobic interactions are likely to alter active‐site dynamics and enhance the stabilization of bulky ligands, thereby improving inhibitory efficiency.

Furthermore, many of the investigated compounds interact with residues lining the access tunnel to the active site, creating a hydrophobic environment that may hinder substrate diffusion. Notably, catechol, verbascoside, and rutin establish specific interactions with ALA A:37, ALA A:80, and LEU A:838, respectively, while other ligands predominantly interact with VAL A:744, VAL A:36, and ALA A:16. Myricetin is distinguished by its simultaneous interaction with VAL A:744, VAL A:36, and ALA A:16, whereas most other compounds form fewer contacts within the tunnel. These interactions are likely to facilitate optimal ligand orientation and stabilization prior to entry into the catalytic cavity, reinforcing their inhibitory effect [36].

Finally, the docking results indicate that phenolic compounds can modulate urease flexibility and conformational dynamics through interactions with residues located both in the catalytic pocket and along the access channel [37, 38]. Residues such as THR A:740, THR A:578, SER A:421, and THR A:33 are recurrently involved in ligand binding, depending on the chemical structure of the inhibitor. These interactions may induce local conformational rearrangements that restrict enzyme flexibility, limit substrate access, or perturb catalytic residue orientation. Among the investigated ligands, salicin appears to exert the most pronounced conformational influence, interacting with three residues implicated in enzyme flexibility. Overall, these findings support a multifactorial inhibition mechanism in which hydrogen bonding, electrostatic interactions, aromatic stacking, and tunnel blocking collectively contribute to urease inhibition by phenolic compounds.

The comparative analysis of phenolic compounds on AChE and urease reveals interesting differences and similarities in their affinity and modes of interaction. Overall, flavonoids and polyphenols such as hesperidin, verbascoside, silymarin, and catechin exhibit the most favorable binding energies for both enzymes, suggesting a significant inhibitory potential. Hesperidin stands out in particular, with a binding energy of –10.2 kcal/mol for AChE and –9.6 kcal/mol for urease, indicating strong stabilization within their respective active sites. The compounds also display a variable number of hydrogen bonds, which play a key role in ligand stabilization. For example, verbascoside forms up to seven hydrogen bonds with each enzyme, whereas other flavonoids such as kaempferol or naringenin form fewer, despite sometimes comparable affinity. Notably, some compounds, including rutin and hesperidin, form more hydrogen bonds with urease than with AChE, which may reflect differences in the topology and polarity of the active site cavities of the two enzymes. These observations suggest that, although these polyphenols have inhibitory potential against both targets, the interaction mechanisms and the contribution of hydrogen bonds may vary depending on the enzyme, which is crucial for the design of dual or selective inhibitors.

## 4 Conclusion

This study demonstrated that the use of lactic acid as a green solvent represents an effective alternative for the extraction of phytochemical compounds from *P. lentiscus* leaves. The obtained extract, rich in bioactive compounds, exhibited strong inhibitory activity against acetylcholinesterase and urease, likely due to the presence of phenolic acids, flavonoids, and coumarins contributing to enzymatic activity. Furthermore, molecular docking analysis highlighted specific interactions of these compounds with key residues of the enzymes, reinforcing their inhibitory potential. These results suggest that the lactic acid extract could serve as a promising approach for the exploitation of bioactive compounds from *P. lentiscus* for pharmacological purpose.

## Author Contributions

**Sidali Zaidi**: manipulation, analysis and interpretation, writing of the manuscript. **Naima Saidene**: experimental handling. **Radia Kadi**: experimental handling. **Nadjet Benaida‐Debbache**: writing of the manuscript.

## Conflicts of Interest

The authors declare no conflicts of interest.

## Supporting information




**Supporting File**: cbdv71831‐sup‐0001‐SuppMat.docx

## Data Availability

The datasets used and/or analyzed during the current study are available from the corresponding author on reasonable request.
