# Supplementary material for: Green Extraction of Phenolic Compounds From Pistacia lentiscus Leaves Coupled With LC‐MS Characterization and Structure‐Based Enzyme Inhibition Analysis
Source: Chem Biodivers. 2026 Oct 12;23(10):e71831. doi: 10.1002/cbdv.71831 (PMC13655636; doi:10.1002/cbdv.71831)
Supplement: Supplementary file 1 [file CBDV-23-e71831-s001.docx]

**Supplementary Material :** HPLC Chromatographic Profile of Phenolic Compounds in *Pistacia lentiscus*

**Figure S1.** Chromatographic profile of phenolic compounds in *Pistacia lentiscus*
